# Iatrogenic genito-urinary fistula following cesarean birth in nine sub-Saharan African countries: a retrospective review

**DOI:** 10.1186/s12884-022-04774-0

**Published:** 2022-07-05

**Authors:** Carrie J. Ngongo, Thomas J. I. P. Raassen, Marietta Mahendeka, Ladeisha Lombard, Jos van Roosmalen

**Affiliations:** 1 RTI International, Global Health Division, Seattle, USA; 2 Independent consultant, Nairobi, Kenya; 3 Bugando Medical Centre, Mwanza, Tanzania; 4 Independent consultant, Cape Town, South Africa; 5 Leiden University Medical Centre and Athena Institute VU University, Amsterdam, Netherlands

**Keywords:** Cesarean birth, Surgery, Quality, Safety, Obstetric fistula, Accident, Iatrogenic, Error

## Abstract

**Background:**

Genito-urinary fistulas may occur as complications of obstetric surgery. Location and circumstances can indicate iatrogenic origin as opposed to pressure necrosis following prolonged, obstructed labor.

**Methods:**

This retrospective review focuses on 787 women with iatrogenic genito-urinary fistulas among 2942 women who developed fistulas after cesarean birth between 1994 and 2017. They are a subset of 5469 women who sought obstetric fistula repair between 1994 and 2017 in Tanzania, Uganda, Kenya, Malawi, Rwanda, Somalia, South Sudan, Zambia, and Ethiopia. We compared genito-urinary fistula classifications following vaginal birth to classifications following cesarean birth. We assessed whether and how the proportion of iatrogenic genito-urinary fistula was changing over time among women with fistula, comparing women with iatrogenic fistulas to women with fistulas attributable to pressure necrosis. We used mixed effects logistic regression to model the rise in iatrogenic fistula among births resulting in fistula and specifically among cesarean births resulting in fistula.

**Results:**

Over one-quarter of women with fistula following cesarean birth (26.8%, 787/2942) had an injury caused by surgery rather than pressure necrosis due to prolonged, obstructed labor. Controlling for age, parity, and previous abdominal surgery, the odds of iatrogenic origin nearly doubled over time among all births resulting in fistula (aOR 1.94; 95% CI 1.48–2.54) and rose by 37% among cesarean births resulting in fistula (aOR 1.37; 95% CI 1.02–1.83). In Kenya and Rwanda the rise of iatrogenic injury outpaced the increasing frequency of cesarean birth.

**Conclusions:**

Despite the strong association between obstetric fistula and prolonged, obstructed labor, more than a quarter of women with fistula after cesarean birth had injuries due to surgical complications rather than pressure necrosis. Risks of iatrogenic fistula during cesarean birth reinforce the importance of appropriate labor management and cesarean decision-making. Rising numbers of iatrogenic fistulas signal a quality crisis in emergency obstetric care. Unaddressed, the impact of this problem will grow as cesarean births become more common.

**Supplementary Information:**

The online version contains supplementary material available at 10.1186/s12884-022-04774-0.

## Background

Labor monitoring and emergency obstetric care prevent maternal and newborn deaths from obstetric complications like prolonged, obstructed labor. Access to surgery is increasing rapidly in low- and middle-income countries, yet infrastructure and staffing may not be in place, particularly in remote areas. Shortages of trained staff, inadequate equipment and supplies, and limited accountability add up to poor working conditions and low staff morale [1]. Maternal and perinatal deaths following cesarean birth are fifty times higher in sub-Saharan Africa than in high-income countries [2].

Accidents during pelvic surgeries can result in injury to the bladder, ureters, vagina or intestines. An example is iatrogenic genito-urinary fistula, an abnormal communication between the bladder or ureter and the uterus/cervix/vagina.

A substantial proportion of fistula repairs are for obstetric fistula, resulting when cephalopelvic disproportion, malpresentation, or malposition led to prolonged, obstructed labor. Without monitoring progress of labor and emergency obstetric care, obstruction can cause fetal and maternal death, pressure necrosis of the bladder and fistula formation.

Because fistula etiology is not always clear, we previously proposed a categorization of iatrogenic fistulas based on anatomical location and circumstances [3]. There is growing concern that cesarean births are the most important cause of iatrogenic fistulas in sub-Saharan Africa [4]. Reports indicate that between 77 and 80.2% of iatrogenic fistulas follow surgery for obstetric complications, whether cesarean section, uterine rupture repair or hysterectomy [3, 5].

Evidence has been mounting about the proportion of iatrogenic fistula amongst repaired fistulas, though with some variation in definitions. Applying our conceptual categorization we previously reported that 13.2% of fistulas in 5959 women were of iatrogenic origin [3]. With funding from the United States Agency for International Development (USAID), EngenderHealth’s multi-country Fistula Care+ Project found that iatrogenic injury accounted for 18% of the fistulas where the surgeon noted etiology [4]. In Niger 9.9% of 624 fistulas were iatrogenic, [6] while in the D.R. Congo 19.4% of 1984 fistulas were non-obstetric, with iatrogenic fistulas accounting for an unspecified share [5]. In Ethiopia, a review of 2593 women with fistula found that 24.6% had “high bladder fistula (predominantly after surgery).” [7] High fistula similarly accounted for 26.3% (119/452) of vesico-vaginal fistulas in Malawi, “likely due to operative injury rather than obstructed labor.” [8] Among 229 women with fistula following cesarean birth in the D.R.Congo, 24.0% had iatrogenic injuries [9]. Overall, the proportion of iatrogenic fistulas appears to be on the rise in low- and middle-income countries [10].

This paper documents fistula classifications among women seeking fistula repair who gave birth between 1994 and 2017 by type of birth (vaginal or cesarean). We hypothesize that iatrogenic fistulas are growing more common over time given rising frequencies of cesarean birth among women who developed fistula.

## Methods

This retrospective record review evaluated frequencies of cesarean-associated iatrogenic fistulas over time amongst women presenting with fistula-related incontinence in places where access to quality emergency obstetric care is often difficult. Women seeking fistula repair were interviewed in 86 facilities in Tanzania, Uganda, Kenya, Malawi, Rwanda, Somalia, South Sudan, Zambia and Ethiopia. Women consented to fistula repair in their respective hospitals, following each hospital’s counseling and informed consent process. Their births took place in an unknown number of hospitals that provided basic and comprehensive emergency obstetric care. Data were collected by the second and third author and their colleagues between June 1994 and December 2017.

Women seeking fistula repair in the nine countries were eligible for inclusion in this analysis if they had a fistula following vaginal birth, cesarean birth, cesarean hysterectomy or uterine rupture repair. Women were excluded if they developed fistulas following gynecological surgery, traumatic injury (from accidents, traditional healers, or sexual violence), abortion, or radiation. We excluded women with perineal tears who did not have a concurrent obstetric vesico-vaginal fistula.

One of the surgeons interviewed the women and recorded information on a standard form, documenting women’s age at presentation, age at fistula development, obstetric history (including previous births and previous laparotomies), and whether the causative birth was vaginal, cesarean, cesarean hysterectomy or uterine rupture repair [3]. Women provided information about the sex and condition of the newborn at birth. In case of multiples, births were counted “alive” if at least one baby was alive at birth. The operating surgeon noted the fistula’s classification based on Waaldijk’s classification system [11]. This categorizes fistulas based on their anatomic/physiologic position, involvement of the closing mechanism and urethra, and whether damage is circumferential. The second author (TR) confirmed classifications for all records to ensure consistency. Apart from information on fistula classifications, our unit of analysis was births.

“Cesarean birth” includes cesarean section, cesarean hysterectomy and uterine rupture repair. Some women had more than one fistula. In these cases, their births were associated with iatrogenic fistula if at least one of the woman’s fistulas was iatrogenic.

Data were entered into an Excel database, with names changed to unique identification numbers to protect privacy. Data were analyzed using Stata 16. Approval for this record review was granted by the AMREF Ethics and Scientific Review Committee (AMREF-ESRC P88/2013).

Fistulas range along a spectrum of certainty from “definitely”, “probably” and “likely” iatrogenic to “not iatrogenic.” [3] Ureteric injuries are definitely iatrogenic, as well as vesico-[utero]/−cervico-vaginal fistulas that occurred with a live birth, given that cesarean birth of a live baby is rarely associated with pressure necrosis [3, 12]. Vault fistulas are probably iatrogenic, as they occur after cesarean hysterectomy or emergency hysterectomy for uterine rupture. Vault fistulas should not be counted as iatrogenic, however, when uterine rupture involved the bladder. Finally, vesico-[utero]/−cervico-vaginal fistulas following cesarean birth with stillbirth are likely iatrogenic when the fistula is clearly in the cervical canal, less than 3 cm, and without bladder rupture. Uncommonly, a vesico-[utero]/−cervico-vaginal fistula will be considered iatrogenic when the woman has a concurrent fistula in a different location. The second author (TR) applied these criteria to determine the origin of fistulas for all included records on the basis of his examination and surgical notes. For readers interested in more conservative definitions, we provide regression outputs for three different outcomes.

Mixed effects logistic regressions modelled the rise in iatrogenic fistula among all births resulting in fistula and among cesarean births resulting in fistula. Models included fixed effects for the year of fistula development, age at fistula development, parity and previous abdominal surgery (nearly always cesarean birth). The models included country of fistula repair as a random effect. We used categorical date ranges so as not to assume a linear change over time (1994–1999 as reference, 2000–2004, 2005–2009, and 2010–2017). Parities with similar effects were consolidated to reduce the number of categories (1, 2, 3–5, 6+). Age at fistula development was likewise grouped (11–19 as reference, 20–24, 25–29, 30–34, 35+). The threshold for statistical significance was *p* < 0.05.

We conducted country-specific logistic regressions for countries with at least 40 women with iatrogenic fistula among at least 300 birth records. Country-specific models controlled for the same variables as above. To contextualize the proportion of iatrogenic fistula within rising frequencies of cesarean birth among women with fistula, we conducted country-specific logistic regressions with an outcome of cesarean birth and the same predictors of year of fistula development, age at development, parity and previous abdominal surgery [13]. Holding other covariates constant, we estimated the proportion of births with fistula that were cesarean births and the proportion of births with fistula resulting in iatrogenic fistula at each time period (1994–1999, 2000–2004, 2005–2009, 2010–2017). We then graphed the fitted values of our country-specific models for both cesarean births and iatrogenic fistula.

## Results

Among 5469 women who sought fistula repair, 2942 (53.8%) reported that they had given birth by cesarean. The remaining 2527 women (46.2%) developed fistula following vaginal birth. Although most women developed one fistula, 531/5469 (9.7%) had multiple fistulas, for a total of 6000 fistulas following 5469 births. Most cesarean births in this series were emergency procedures. Only six women had elective cesarean birth without labor.

Table 1 provides frequencies by Waaldijk’s classification for all fistulas in the dataset. According to this classification Type I vesico-cervico-vaginal fistula, Type I vault fistula, and Type III ureteric injuries following cesarean birth generally do not occur in women who gave birth vaginally. Two women did have iatrogenic injuries after vaginal birth: one after vacuum extraction and another after postpartum dilatation and curettage. Anatomical location and circumstances indicated that 508/3158 (16.1%) of fistulas following cesarean birth were definitely iatrogenic, 95/3158 (3.0%) were probably iatrogenic, and 194/3158 (6.1%) were likely iatrogenic. Over one-quarter of fistulas following cesarean birth were iatrogenic (796/3158, 25.2%). Given that some women had multiple fistulas, the share was higher among women: 787/2942 (26.8%) of women with fistula after cesarean birth had an iatrogenic fistula rather than a fistula due to pressure necrosis of the bladder.

**Table 1:**
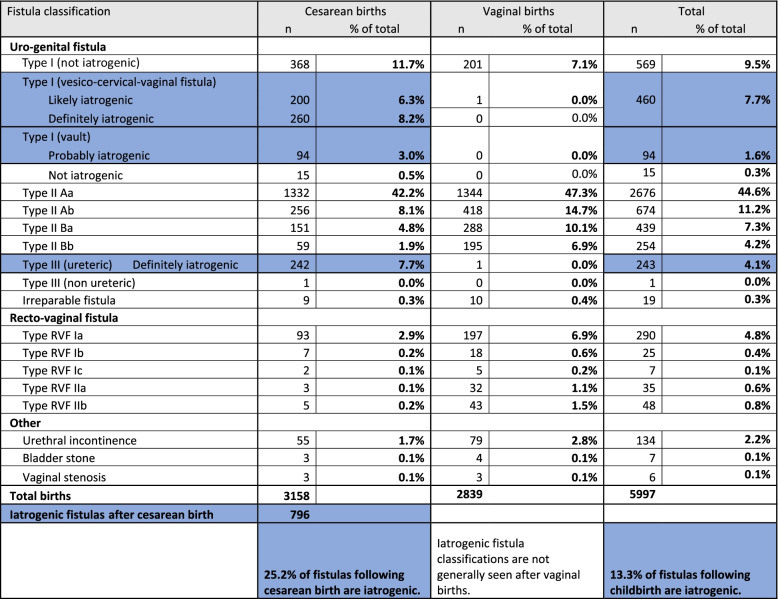
Waaldijk’s fistula classifications following cesarean and vaginal birth

While cesarean birth rates have changed over time in births leading to fistula, on average just over half of women with fistula reported cesarean births in Malawi (321/560, 57.3%), Kenya (475/868, 54.7%), Rwanda (192/3601, 53.32%), and Tanzania (969/1838, 52.7%) [13]. Somalia had the lowest share of women with fistula reporting cesarean birth (69/260, 26.5%). Cesarean birth was more common in women with fistula in Zambia (84/135, 62.2%) and Uganda (741/1161, 63.8%) (Table S1).

Consolidated data from the 23-year period indicated that iatrogenic fistula following cesarean birth was more common in Uganda (224/741, 30.2%) and Kenya (137/475, 28.8%), followed by Rwanda (50/192, 26.0%) and Malawi (83/321, 25.9%). The proportion of iatrogenic fistulas among fistulas after cesarean birth was lowest in South Sudan (15/76, 19.7%) and Somalia (10/69, 14.5%) (Table S1).

Controlling for age, parity and previous abdominal surgery, the odds of iatrogenic origin doubled between 1994 and 2017 among all births resulting in fistula (aOR 1.94; 95% CI 1.48–2.54) (Table 2). Odds of iatrogenic fistula tripled in women who reported previous abdominal surgery (aOR 3.00; 95% CI 2.41–3.73). Odds of iatrogenic fistula increased with rising parity: aOR 2.60 (95% CI 1.92–3.53) at para 2, aOR 2.98 (95% CI 2.18–4.07) at para 3–5, and aOR 4.80 (95% CI 3.32–6.94) at para 6 or more. Women over age 25 developed iatrogenic fistulas more often than younger women and girls, with aOR ranging from 2.46 (95% CI 1.62–3.75) to 2.64 (95% CI 1.78–3.93). Effects were similar across definitions of iatrogenic fistulas.

**Table 2:** Mixed effects logistic regression of iatrogenic fistula among all births with fistula

		Likely iatrogenic^**a**^ (“likely” + “probably” + “definitely”)			Probably iatrogenic^**b**^ (“probably” + “definitely”)			Definitely iatrogenic^**c**^ (“definitely”)		
		*n* = 791			*n* = 618			*n* = 524		
Fixed-effect covariates^d^	n	Odds ratio	95% confidence interval		Odds ratio	95% confidence interval		Odds ratio	95% confidence interval	
Date of fistula development										
1994–1999	1478	(Ref)	(Ref)		(Ref)	(Ref)		(Ref)	(Ref)	
2000–2004	1870	1.21	0.96	1.52	1.25	0.97	1.61	1.42	1.08	1.88
2005–2009	1211	1.56	1.22	2.00	1.72	1.31	2.25	1.99	1.49	2.67
2010–2017	910	1.94	1.48	2.54	1.90	1.41	2.56	2.20	1.60	3.04
Previous abdominal surgery										
No	4965	(Ref)	(Ref)		(Ref)	(Ref)		(Ref)	(Ref)	
Yes	501	3.00	2.41	3.73	2.81	2.23	3.53	3.38	2.66	4.30
Parity										
1	2762	(Ref)	(Ref)		(Ref)	(Ref)		(Ref)	(Ref)	
2	721	2.60	1.92	3.53	2.31	1.63	3.26	2.06	1.43	2.97
3–5	1177	2.98	2.18	4.07	2.82	1.99	3.99	2.37	1.64	3.44
6+	805	4.80	3.32	6.94	4.50	3.01	6.75	3.77	2.44	5.82
Age at fistula development										
11–19	2020	(Ref)	(Ref)		(Ref)	(Ref)		(Ref)	(Ref)	
20–24	1422	1.79	1.31	2.44	2.02	1.42	2.87	2.04	1.41	2.94
25–29	874	2.62	1.84	3.74	2.82	1.89	4.22	2.62	1.72	4.01
30–34	557	2.64	1.78	3.93	2.91	1.87	4.54	2.59	1.61	4.16
35+	583	2.46	1.62	3.75	2.56	1.60	4.09	2.46	1.49	4.05
Random-effect covariate										
Country	5469	0.20	0.06	0.65	0.15	0.04	0.56	0.27	0.08	0.90

^a^“Likely iatrogenic” includes: Type III ureteric injuries, Type I VCVF with live baby, Type I vault or Type I VCVF in women with clear obstetric fistula in another location, Type I vault fistula, and Type I VCVF with stillbirth

^b^“Probably iatrogenic” includes: Type III ureteric injuries, Type I VCVF with live baby, Type I vault or Type I VCVF in women with clear obstetric fistula in another location, and Type I vault fistula

^c^“Definitely iatrogenic” includes: Type III ureteric injuries, Type I VCVF with live baby, and Type I vault or Type I VCVF in women with clear obstetric fistula in another location

^d^Observations used in regression = 5451

Rising frequencies of cesarean as a mode of birth accounted for some of the observed increase in iatrogenic fistula. Limiting regression to women who developed fistula after cesarean birth, iatrogenic fistula rose by 37% in 2010–2017 as compared to 1994–1999 (aOR 1.37; 95% CI 1.02–1.83) (Table 3). Odds of iatrogenic fistula were 146% higher in women with previous abdominal surgery (aOR 2.46; 95% CI 1.93–3.13). As seen with all births resulting in fistula, the odds of iatrogenic fistula increased with rising parity: aOR 2.12 (95% CI 1.53–2.95) at para 2, aOR 2.18 (95% 1.55–3.05) at para 3–5, and OR 3.17 (95% CI 2.12–4.76) at para 6 or more. Effects were similar across iatrogenic fistula categories.

**Table 3:** Mixed effects logistic regression of iatrogenic fistula among cesarean births with fistula

		Likely iatrogenic^**a**^ (“likely” + “probably” + “definitely”)			Probably iatrogenic^**b**^ (“probably” + “definitely”)			Definitely iatrogenic^**c**^ (“definitely”)		
		*n* = 787			*n* = 616			*n* = 522		
Fixed-effect covariates^d^	n	Odds ratio	95% confidence interval		Odds ratio	95% confidence interval		Odds ratio	95% confidence interval	
Date of fistula development										
1994–1999	685	(Ref)	(Ref)		(Ref)	(Ref)		(Ref)	(Ref)	
2000–2004	965	1.05	0.81	1.34	1.09	0.83	1.43	1.26	0.94	1.69
2005–2009	714	1.24	0.95	1.62	1.39	1.04	1.85	1.63	1.20	2.21
2010–2017	578	1.37	1.02	1.83	1.36	1.00	1.85	1.60	1.14	2.24
Previous abdominal surgery										
No	2541	(Ref)	(Ref)		(Ref)	(Ref)		(Ref)	(Ref)	
Yes	398	2.46	1.93	3.13	2.26	1.76	2.89	2.77	2.14	3.57
Parity										
1	1187	(Ref)	(Ref)		(Ref)	(Ref)		(Ref)	(Ref)	
2	429	2.12	1.53	2.95	1.83	1.27	2.62	1.61	1.10	2.36
3–5	753	2.18	1.55	3.05	2.05	1.42	2.97	1.70	1.15	2.52
6+	570	3.17	2.12	4.76	2.98	1.93	4.59	2.45	1.54	3.88
Age at fistula development										
11–19	931	(Ref)	(Ref)		(Ref)	(Ref)		(Ref)	(Ref)	
20–24	701	2.04	1.47	2.81	2.26	1.57	3.25	2.26	1.55	3.30
25–29	551	2.93	2.01	4.28	3.00	1.97	4.58	2.76	1.77	4.30
30–34	373	3.12	2.04	4.79	3.26	2.04	5.21	2.85	1.73	4.68
35+	379	3.14	1.99	4.96	3.02	1.83	4.98	2.88	1.69	4.88
Random-effect covariate										
Country	2942	0.05	0.01	0.28	0.04	0.00	0.25	0.10	0.02	0.43

^a^“Likely iatrogenic” includes: Type III ureteric injuries, Type I VCVF with live baby, Type I vault or Type I VCVF in women with clear obstetric fistula in another location, Type I vault fistula, and Type I VCVF with stillbirth

^b^“Probably iatrogenic” includes: Type III ureteric injuries, Type I VCVF with live baby, Type I vault or Type I VCVF in women with clear obstetric fistula in another location, and Type I vault fistula

^c^“Definitely iatrogenic” includes: Type III ureteric injuries, Type I VCVF with live baby, and Type I vault or Type I VCVF in women with clear obstetric fistula in another location

^d^Observations used in regression = 2931

**Fig. 1:**
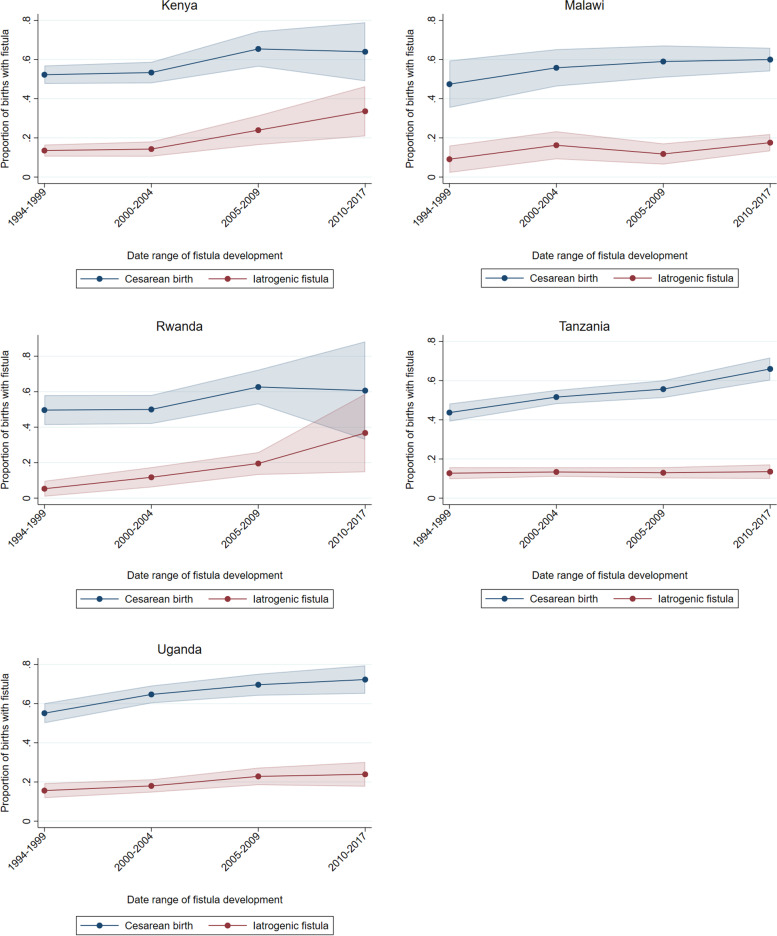
Rise in cesarean birth and iatrogenic injury among women with fistula

Country-specific logistic regressions revealed correlations between iatrogenic fistula and rising cesarean birth rates among women who developed fistulas in Kenya, Malawi, Rwanda, Tanzania, and Uganda (Fig. 1). Cesarean birth as mode of birth grew more frequent in all countries in women who developed fistulas after birth. In Malawi and particularly Tanzania, the proportion of iatrogenic fistula rose less than the increase in cesarean birth rates reported by women seeking fistula repair. In Uganda the rise in iatrogenic fistula roughly matched the rise in cesarean birth. In Kenya and Rwanda, the increase in iatrogenic fistula outpaced the rise in cesarean birth.

## Discussion

Despite its important place in enabling fistula elimination, cesarean birth does not always prevent fistula from occurring after prolonged, obstructed labor. Over 23 years iatrogenic fistula rose substantially in this population of 5469 African women seeking obstetric fistula repair, revealing gaps in the quality of obstetric surgery offered to marginalized women. These women suffer needlessly, as iatrogenic injury during obstetric surgery is preventable with quality emergency obstetric care.

Cesarean birth is increasingly common, including among women with obstructed labor and intrauterine fetal death [13] and generally at population level [14]. Rising numbers of cesarean births increase the risk of all associated complications, including iatrogenic fistula. The seriousness of cesarean birth complications in resource-limited settings reinforces the importance of appropriate labor management and restrained cesarean decision-making [15]. Cesarean birth should only be performed when clear anticipated benefits outweigh the additional risks and higher costs [15, 16].

Rising cesarean birth rates mean more opportunities for surgical complications. One might therefore expect that the proportion of iatrogenic fistulas will rise among all births resulting in fistula. Our comparison between the odds of iatrogenic fistula development amongst all births resulting in fistula and amongst cesarean births resulting in fistula reveals that the issue cannot be explained solely by cesarean birth rates. In some countries, cesarean birth has grown not only more frequent, but also more hazardous.

Women with obstructed labor should be able to quickly access health facilities with staffing, equipment and infrastructure to provide high-quality basic and comprehensive emergency obstetric care. One report found that nearly three-quarters of women with fistula presented to a hospital or health center during early labor [17]. The fact that these women went on to develop fistula indicates serious deficiencies in care, facilities and referral systems.

Basic facility readiness cannot be assumed. Only one-third of facilities offering cesarean birth in Tanzania demonstrated readiness by having consistent electricity, 24-hour staff availability and general anesthesia equipment [18]. Health facilities without steady electricity, piped water, dedicated space and basic equipment could hardly be considered appropriate work environments.

These readiness considerations are minimum requirements for quality maternity care. Indeed, physical and human resources are just two of the eight standards that the World Health Organization (WHO) has proposed to improve the quality of maternal and newborn care in facilities [19]. Some standards relate to provision of care: Healthcare providers should employ evidence-based practices for routine care and management of complications. They should have actionable information systems and functional referral systems. Other standards connect to women’s experience of care: respect, emotional support and effective and responsive communication [20].

Quality maternity care hinges on healthcare provider training, mentoring, and supervision. Healthcare providers should recognize signs of complicated labor progression and make appropriate decisions about referral. Clear management guidelines with partograph use can improve outcomes [21, 22]. Unfortunately, staffing and capacity gaps and other constraints have led to inadequate partograph use, and new WHO guidance on the partograph runs the risk of confusion leading to more prolonged labours ending in cesarean section, risking surgical complications such as iatrogenic fistula [22, 23]. Training should emphasize the decision-making process that leads to cesarean birth, ensuring that cesareans are performed only when indicated. Alternative modes of birth should remain in medical curricula, including vacuum extraction for prolonged second stage or foetal distress and craniotomy for intrauterine fetal death resulting from obstructed labor [13, 24, 25]. Training is a central challenge, as familiarity with craniotomy and vacuum extraction is rarer than knowledge of cesarean birth. Training should highlight optimal operative techniques [3, 25].

Women with past abdominal surgery have a heightened risk of iatrogenic fistula, as scar tissue and adhesions can create challenges for healthcare providers performing cesarean section [3]. Healthcare providers should recognize the increased risk for women with previous cesarean birth and proceed with caution, by making the incision above the vesico-uterine peritoneal fold without creating a bladder flap [26].

Our analysis found that the most dramatic increase in iatrogenic fistula risk over time occurred in Rwanda and Kenya. This contrasts with Tanzania and Malawi, two countries where associate clinicians (assistant medical officers and clinical officers) perform cesarean section. Although we did not find evidence that iatrogenic fistula risk is rising over time in Tanzania and Malawi, the proportion of iatrogenic fistulas following cesarean birth steadily remained at around one-quarter of women with fistula after cesarean birth in these two countries. Geospatial differences can invite future investigations into possible differences in training, supervision, and infrastructure between countries.

### Limitations

This large, multi-country retrospective review is not without limitations. All women in this study had developed fistulas, precluding comparison with women who died from unattended obstructed labor or who were spared a fistula thanks to appropriate management of prolonged labor and timely cesarean birth. Women with fistula who seek treatment may differ from those who do not. Country sample size varied by where the second and third author and colleagues conducted repairs. We do not have a reason to doubt that the women in our review were representative of all women seeking fistula treatment, although selection bias is possible. We minimized variability by having a single surgeon confirm fistula classifications.

We relied on information from women about past events. In many cases, years passed between the day of birth and when women presented for fistula repair. The validity of self-reported data has been examined with mixed results [27, 28]. We acknowledge that women’s recollection of childbirth may differ from how healthcare providers diagnose obstetric problems. We recognize that retrospective studies face inherent limitations, such that inferences focus on associations.

Women seek fistula treatment after fistula development, with variable durations of incontinence. Our period of focus aligns the start date of data collection with the year of fistula development: to avoid bias we excluded earlier-occurring fistulas that were repaired in or after 1994. At the end of our study period, however, it is possible that long-duration fistulas would be underrepresented, leading to higher iatrogenic fistula estimates since women with iatrogenic fistula are more likely to receive treatment earlier than women with obstetric fistula following prolonged, obstructed labor [3].

## Conclusions

Reducing the incidence of iatrogenic injuries in obstetric surgery will require a holistic approach to health system functioning, addressing current gaps in infrastructure, staffing, communications, access to drugs, surgical material and equipment. Future occurrence of iatrogenic fistula can be minimized through surgical training, adequate supervision, and audit of cases to learn lessons. Because iatrogenic fistulas occur, high-level facilities offering fistula repair are advised to treat iatrogenic fistula as a sentinel event. Documentation and follow-up with relevant facilities will raise awareness and spur local quality improvement measures. Healthcare providers should follow evidence-based guidelines for labor management and cesarean decision-making, recognizing situations that call for alternatives to cesarean birth. Preventing unnecessary surgeries will prevent iatrogenic fistula, reducing suffering among the most vulnerable.

## Supplementary Information

**Additional file 1: Table S1.** Iatrogenic fistula and cesarean births by country.

## Data Availability

Data generated and analysed during the current study are not publicly available during a period of analysis and dissemination but will be available from the corresponding author on reasonable request.
