# Supplementary material for: Iatrogenic genito-urinary fistula following cesarean birth in nine sub-Saharan African countries: a retrospective review
Source: BMC Pregnancy Childbirth. 2022 Jul 5;22:541. doi: 10.1186/s12884-022-04774-0 (PMC9254569; doi:10.1186/s12884-022-04774-0)
Supplement: Supplementary file 1 — Additional file 1: Table S1. Iatrogenic fistula and cesarean births by country. [file 12884_2022_4774_MOESM1_ESM.pdf]

Table S1: Iatrogenic fistula and cesarean births by country

Country	Births				Cesarean births resulting in iatrogenic fistula	
	Total	Vaginal	Cesarean			
	N	N	N	%	N	%
Ethiopia	89	75	14		6	
Kenya	868	393	475	54.7%	137	28.8%
Malawi	560	239	321	57.3%	83	25.9%
Rwanda	360	168	192	53.3%	50	26.0%
Somalia	260	191	69	26.5%	10	
South Sudan	198	122	76	38.4%	15	
Tanzania	1838	869	969	52.7%	242	25.0%
Uganda	1161	420	741	63.8%	224	30.2%
Zambia	135	51	84	62.2%	20	
Total	5469	2528	2941	53.8%	787	26.8%
